# Efficacy of IOTA simple rules, O-RADS, and CA125 to distinguish benign and malignant adnexal masses

**DOI:** 10.1186/s13048-022-00947-9

**Published:** 2022-01-23

**Authors:** Wen ting Xie, Yao qin Wang, Zhi sheng Xiang, Zhong shi Du, Shi xin Huang, Yi jie Chen, Li na Tang

**Affiliations:** 1 Department of Ultrasound, Fujian Medical University Cancer Hospital, Fujian Cancer Hospital, Fuzhou, 350014 Fujian Province China; 2 Department of Epidemiology, Fujian Medical University Cancer Hospital, Fujian Cancer Hospital, Fuzhou, 350014 China

**Keywords:** Adnexal masses_1_, Ultrasound_2_, O-RADS_3_, IOTA_4_, Cancer antigen 125_5_, Malignancy risk_6_

## Abstract

**Objective:**

Ovarian cancer is the most deadly deadliest gynecological tumor in the female reproductive system. Therefore, the present study sought to determine the diagnostic performance of International Ovarian Tumor Analysis Simple Rules (IOTA SR), the Ovarian-Adnexal Reporting and Data System (O-RADS), and Cancer Antigen 125 (CA125) in discriminating benign and malignant ovarian tumors. The study also assessed whether a combination of the two ultrasound categories systems and CA125 can improve the diagnostic performance.

**Methods:**

A total of 453 patients diagnosed with ovarian tumors were retrospectively enrolled from Fujian Cancer Hospital between January 2017 and September 2020. The data collected from patients included age, maximum lesion diameter, location, histopathology, levels of CA125, and detailed ultrasound reports. Additionally, all ultrasound images were independently assessed by two ultrasound physicians with more than 5 years of experience in the field, according to the IOTA simple rules and O-RADS guidelines. Furthermore, the area under the curve (AUC), sensitivity, and specificity of the above mentioned predictors were calculated using the receiver operating characteristic curve.

**Results:**

Out of the 453 patients, 184 had benign lesions, while 269 had malignant ovarian tumors. In addition, the AUCs of IOTA SR, O-RADS, and CA125 in the overall population were 0.831, 0.804, and 0.812, respectively, and the sensitivities of IOTA SR, O-RADS, and CA125 were 94.42, 94.42, and 80.30%, respectively. On the other hand, the AUCs of IOTA SR combined with CA125, O-RADS combined with CA125, and IOTA SR plus O-RADS combined with CA125 were 0.900, 0.891, and 0.909, respectively. The findings also showed that the AUCs of a combination of the three approaches were significantly higher than those of individual strategies (*p*<0.05) but not significantly higher than the AUC of a combination of two methods (*p*>0.05).

**Conclusion:**

The findings showed that a combination of IOTA SR or O-RADS in combination with CA125 may improve the ability to distinguish benign from malignant ovarian tumors.

**Supplementary Information:**

The online version contains supplementary material available at 10.1186/s13048-022-00947-9.

## Introduction

Ovarian cancer is one of the deadliest gynecological tumors and one of the three most common malignancies in the female reproductive system [15]. In addition, most women with ovarian cancer are diagnosed at an advanced stage due to the lack of early symptoms related to the malignancy [10]. It has also been reported that the 5-year survival rate of patients with late-stage ovarian cancer is < 50% (National Cancer Institute Cancer stat facts: ovarian cancer. Available at: https://seer.cancer. gov/statfacts/html/ovary.html.[Accessed June 7, 2021]). Timely identification of malignant and benign ovarian tumors is therefore essential to guide therapeutic decisions and prognosis.

Ultrasound (US) is a non-invasive imaging method used in the identification of benign and malignant adnexal masses [16]. Additionally, several guidelines and structured reporting based on ultrasound characterization have been proposed to help assess the risk of malignancy in ovarian masses. One such guideline that is extensively used in clinical practice is the International Ovarian Tumor Analysis (IOTA) Simple Rules (SR), which was proposed by the International Ovarian Tumor Analysis group in 200 8[17]. IOTA SR consists of five US features of benign tumors and five US features that can be used to identify malignant tumors. Additionally, the Ovarian-Adnexal Reporting and Data System (O-RADS) was published by the American College of Radiology (ACR) and provides guidelines for ovarian management in high-risk categories [2].

Moreover, several previous studies reported that US classification systems are highly effective in the detection of malignant ovarian cancer. In addition, IOTA SR has been validated in multiple studies and has been shown to have the highest value in predicting preoperative differentiation of adnexal tumors [18]. IOTA SR is also easy to use, and nonexpert examiners can easily be trained on the technique. This method therefore makes it easy for clinicians to classify adnexal masses because of its simplicity [11]. However, the diagnostic performance of the O-RADS has not been validated; therefore, its utilization still needs to be verified.

Tumor biomarkers also play a vital role in the preoperative detection of ovarian cancer. Notably, cancer antigen 125 (CA125) is the most promising and significant marker in the screening, detection, and monitoring of ovarian cancer [12]. However, serum levels of CA125 have been shown to be increased not only in ovarian cancer but also in other pathological conditions or benign diseases such as pregnancy and endometriosis. Additionally, the levels of CA125 vary in different subtypes of ovarian cancer [7]. The use of CA125 alone in the diagnosis of ovarian cancer is therefore insufficient.

Furthermore, the use of ultrasound alone or in combination with serum tumor biomarkers has been shown to be a better method of diagnosing adnexal masses. Consequently, the present study aimed to assess the diagnostic performance of IOTA SR, O-RADS and CA125 in discriminating benign from malignant ovarian tumors. The present study also assessed whether a combination of these ultrasound systems with CA125 could improve the diagnostic performance.

## Materials and methods

### Study patients

This retrospective study recruited women with adnexal masses and planned surgical resections in the Fujian Cancer Hospital between January 2017 and September 2020. Patient sociodemographic and clinical characteristics were also collected before surgery from the electronic medical records. In addition, the levels of CA125 were obtained at the time of the preoperative examination. Moreover, histopathological findings from the surgical samples were used as the gold standard and were therefore obtained in all cases. A total of 453 patients were finally enrolled in the study. This retrospective study was approved by the ethical committee of the Fujian Cancer Hospital (SQ2020–013-01, obtained February 11, 2020).

### Ultrasound examination

All the included patients were examined through conventional abdominal gynecological grayscale and color Doppler ultrasonography or transvaginal ultrasound using ultrasound equipment. Additionally, sonographic examination of ovarian masses was performed using a Philips Ultrasound iU22, GE Logiq E9 at a frequency of 1–6 MHz for transabdominal sonography and 6 MHz for transvaginal sonography. All ultrasound examinations were performed within 120 days before surgery by experienced radiologists, and all images were saved in the archiving and communication systems (PACS) of Fujian Cancer Hospital.

### Retrospective images analysis

In the event of bilateral ovarian masses, the larger mass or mass with the worst morphology was chosen for the analysis. Before image analysis, all the participating radiologists were trained by learning the IOTA SR and O-RADS US classification systems online (http://www.iotagroup.org and https://www.acr.org/Clinical-Resources/Reporting-and-Data-Systems/O-Rads). All saved US images were then assessed independently by two ultrasound physicians, each with more than 5 years of experience in the field (YQW and SXH reviewed US images by O-RADS categorizing; WTX and ZSD reviewed US images by IOTA SR). The ultrasound physicians had no knowledge of the patients’ histopathological findings and their results, other than the US imaging information. If there was disagreement between the two radiologists, all US images were reassessed in detail until a final agreement was reached. Finally, a consensus review with the radiologists was achieved to reach the final categorization by the IOTA SR and O-RADS US classification systems.

Moreover, the IOTA SR included ten descriptors that were grouped into two sets: benign features and malignant features. The benign features included a unilocular cyst (B1); solid component < 7 mm in diameter (B2); presence of acoustic shadows (B3); a smooth multilocular tumor with the largest diameter < 10 cm (B4) and no detectable color Doppler signal (B5). On the other hand, the malignant features included an irregular solid tumor (M1); ascites (M2); 4 papillary structures (M3); an irregular multilocular mass > 10 cm in diameter (M4) and a strong color Doppler signal (M5). If one or more M features was present but the B feature was absent, the mass was considered to be malignant. However, if a mass had one or more B features but no malignant features, it was considered to be benign. Moreover, the findings were regarded as inconclusive if both the B and M features were either present or absent (http://www.iotagroup.org). In this study, patients with inconclusive masses were considered to have malignant tumors.

Based on the O-RADS guidelines, six categories were used for risk classification. These included O-RADS 0 (an incomplete evaluation); O-RADS 1 (the physiologic category, including a normal premenopausal ovary); O-RADS 2 (the almost certainly benign category, < 1% risk of malignancy); O-RADS 3 (low risk of malignancy, 1% -<10%); O-RADS 4 (intermediate risk of malignancy, 10–50%); and O-RADS 5 (high risk of malignancy, ≥50% )[3]..

### Statistical analysis

All the data were analyzed using SPSS software (version 22.0.1, SPSS Inc., Chicago, IL, USA) and MedCalc. Additionally, adnexal masses were classified as either benign or malignant based on the histopathological findings for comparisons. Patient demographic data were also compared between the benign and malignant ovarian tumors using the chi-square test for categorical data and Student’s t-test for continuous data. In addition, the area under the receiver operating characteristic curve (AUC) was determined for IOTA SR, O-RADS and CA 125 alone and in combination. The AUCs of each parameter were then compared to verify their efficiency in differentiating malignant and benign tumors. The best cutoff value for CA125 was also determined using a receiver operating characteristic curve (ROC), and the optimal cutoff reference value was 42.05 U/mL. Furthermore, the accuracy, sensitivity, specificity and positive (PPV) and negative (NPV) predictive values of IOTA SR, O-RADS and CA 125 were calculated.

Kappa statistics were used to assess inter-reader agreement of IOTA SR and O-RADS. The κ values were interpreted as follows: 0.1–0.20 was poor agreement; 0.2–0.40 was fair agreement; 0.41–0.60 was moderate agreement; 0.61–0.80 was good agreement; and 0.81–1.0 was very good agreement. A *p* value < 0.05 was considered to be statistically significant.

## Results

### Demographic data and histological findings in the study

**Fig. 1:**
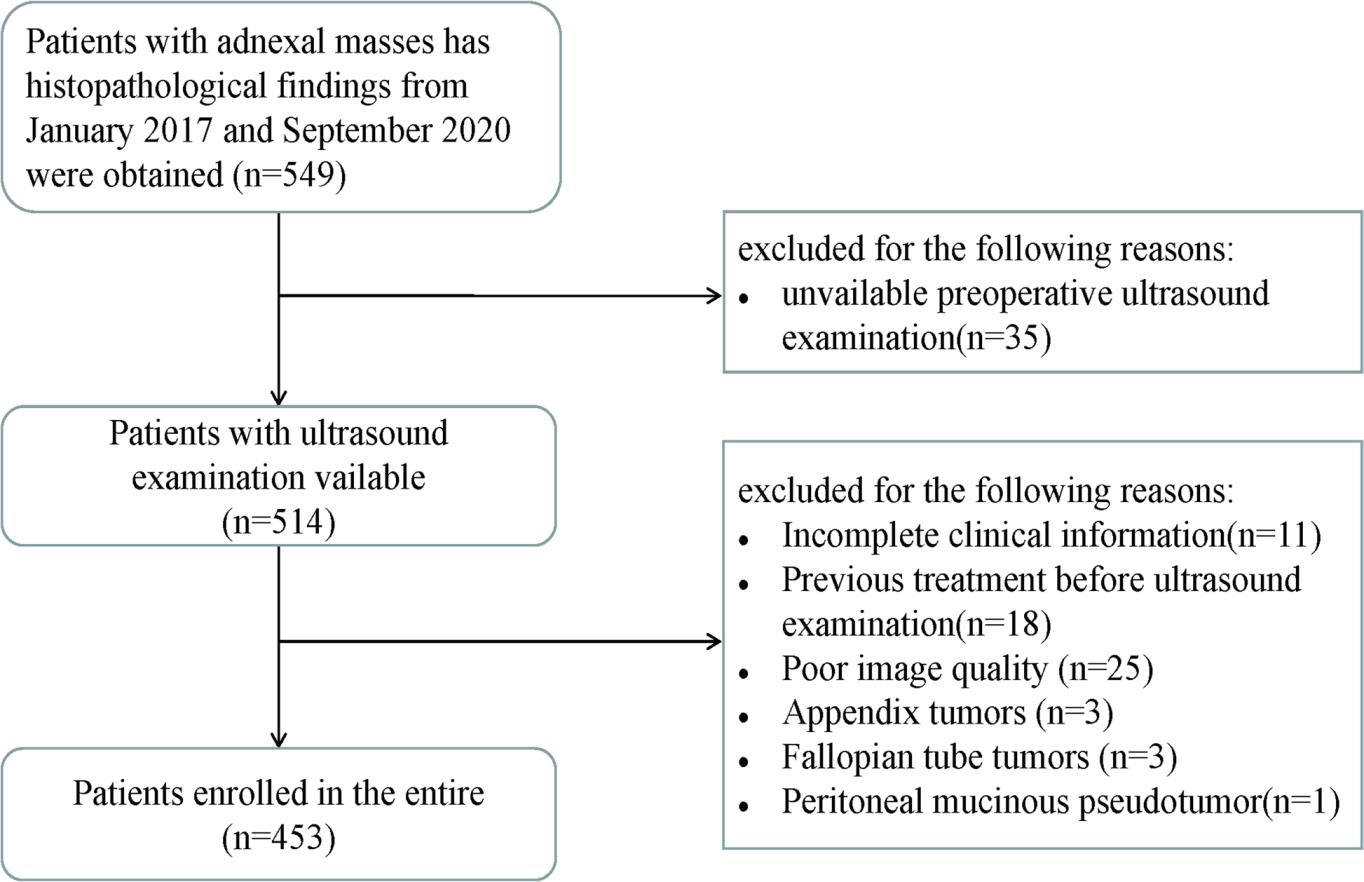
Flow chart of the enrolled patients in present study

The present study included 453 women with adnexal masses; 269 (59.38%) had malignant ovarian tumors, while 84 (40.62%) had benign masses. The final diagnoses are described in Table 1, and the patients enrolled are illustrated as a flowchart in Fig. 1. The findings showed that a majority of the benign ovarian masses were mature teratomas (15.67%), while the malignant tumors were serous cystadenocarcinoma (34.88%).

**Table 1:** Final pathological diagnosis of 453 adnexal masses

Pathologic diagnosis	No.(%)
Benign adnexal masses	184 (40.62)
Mature teratoma	71 (15.67)
Mucinous cystadenoma	49 (10.82)
Serous cystadenoma	31 (6.84)
Thecoma fibroma	8 (1.77)
Struma ovarii	6 (1.32)
Thecoma of the ovary	5 (1.10)
Fibroma	4 (0.88)
Serous adenofibroma	2 (0.44)
Brenner tumor	2 (0.44)
Endometrioid cyst	2 (0.44)
Microcystic stromal tumor	1 (0.22)
Wolffian tumor	1 (0.22)
Inclusion cyst	1 (0.22)
Mucinous adenofibroma	1 (0.22)
Malignant adnexal masses	269 (59.38)
Serous cystadenocarcinoma	158 (34.88)
Borderline mucinous cystadenoma	28 (6.18)
Borderline serous cystadenoma	20 (4.42)
Clear cell carcinoma	15 (3.31)
Metastatic carcinoma	13 (2.87)
Endometrioid carcinoma	6 (1.32)
Malignant Mullerian tube mixed tumor	5 (1.10)
Granular cell tumor	4 (0.88)
Mixed carcinoma	4 (0.88)
Mucinous cystadenocarcinoma	3 (0.66)
Ovarian dysgerminoma	2 (0.44)
Sertoli-Leydig cell tumor	2 (0.44)
Endometrioid borderline tumor	2 (0.44)
Yolk sac tumor	2 (0.44)
Immature teratoma	2 (0.44)
Adult granulose cell tumor of the ovary	1 (0.22)
Borderline Brenner Tumor	1 (0.22)
Small cell carcinoma of the ovary-hypercalcemic type	1 (0.22)

Data are given as n (%)

The patient characteristics are also shown in Table 2. The mean age of the patients in general was 48.75 ± 13.40 (range, 12–81) years, the mean age of patients with benign masses was 45.02 ± 14.97 years, and the mean age of patients with malignant tumors was 51.30 ± 11.59 years (*p*<0.01). In addition, the median tumor diameter was 105.90 ± 64.03 (range, 22–400) mm, benign masses had a mean diameter of 106.17 ± 68.01 mm, and malignant tumors had a mean of 105.36 ± 63.03 mm (*p* > 0.05). In this study, a larger mass was considered if a patient had a bilateral ovarian mass. Moreover, a total of 229 (50.55%) ovarian tumors were located on the left, while 224 (49.45%) tumors were located on the right (*p* > 0.05).

**Table 2:** Patient demographic data and serum CA 125 according to histological findings in the study

All patients	Statistic	Benign Tumor (*n* = 184)	Borderline/Malignant Tumor (*n* = 269)	Total	*p*
Age (years) (Minimum-Maximum)	Mean ± SD	45.02 ± 14.97 (18–78)	51.30 ± 11.59 (12–81)	48.75 ± 13.40 (12–81)	<0.001
Max lesion diameter, mm	Mean ± SD	106.17 ± 68.01 (23–360)	105.36 ± 63.03 (22–400)	105.90 ± 64.03 (22–400)	>0.05
Location	No.(%)				>0.05
left		92/229 (40.17)	137/229 (59.82)	229	
right		92/224 (41.07)	132/224 (58.93)	224	
CA125 (U/mL)	Median	14	231	59	

*CA 125* Cancer Antigen 125

### Distribution of categories in the US classification systems and calculation of malignancy rates

The IOTA SR and O-RADS US classification systems were used to obtain the frequency of the categories presented in Table 3. Classification based on the IOTA SR US system showed that 147 out of the 453 patients (32.45%) had benign masses, 229 (50.55%) had malignant tumors, and 77 (17.00%) of the cases were inconclusive. In addition, the percentages of malignancy in the benign, malignant, and inconclusive ovarian masses were 10.20, 91.27, and 58.44%, respectively (*p*<0.001). On the other hand, the O-RADS guidelines showed that the number of patients in categories 2, 3, 4, and 5 was 78 (17.22%), 59 (13.02%), 134 (29.58%) and 182 (40.18%), respectively. Moreover, the percentages of malignancy in O-RADS 2, 3, 4, and 5 were 5.13, 18.64, 61.19 and 94.50%, respectively (*p*<0.001).

**Table 3:** Comparison of IOTA Simple Rules and O-RADS with histopathological findings and malignancy rates in the categories of two ultrasound classification systems

US Classification Systems	Total No.(%) *n* = 453	Histopathological Result		Calculated malignancy rate (%)	*p*
		Benign *n* = 184	Malignant *n* = 269		
IOTA Simple Rules					<0.001
Benign	147 (32.45)	132 (71.74)	15 (5.57)	10.20	
Malignant	229 (50.55)	20 (10.87)	209 (77.70)	91.27	
Inconclusive	77 (17.00)	32 (17.39)	45 (16.73)	58.44	
O-RADS					<0.001
O-RADS 2	78 (17.22)	74 (40.22)	4 (1.49)	5.13	
O-RADS 3	59 (13.02)	48 (26.09)	11 (4.09)	18.64	
O-RADS 4	134 (29.58)	52 (28.26)	82 (30.48)	61.19	
O-RADS 5	182 (40.18)	10 (5.43)	172 (63.94)	94.50	

*IOTA* International Ovarian Tumor Analysis, *O-RADS* Ovarian Adnexal Reporting and Data System

### Diagnostic performance of CA-125 and the US classification systems

**Fig. 2:**
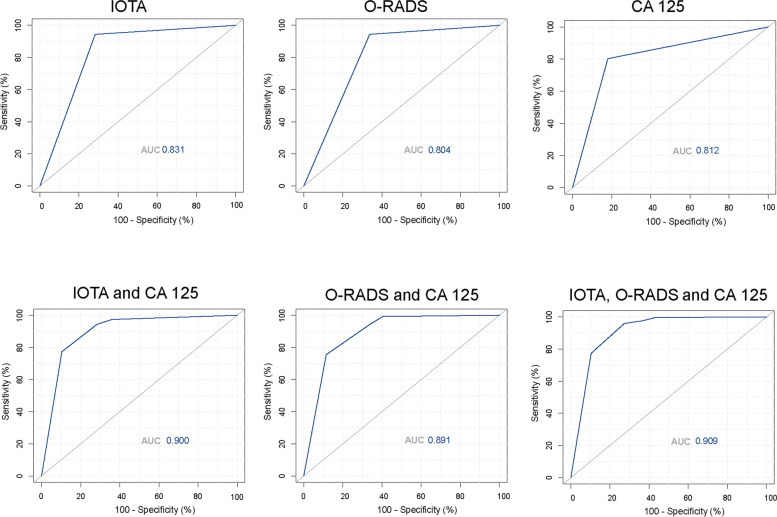
ROC analysis of IOTA, O-RADS and CA 125. ROC = Receiver Operating Characteristic; O-RADS = Ovarian Adnexal Reporting and Data System; IOTA SR = International Ovarian Tumor Analysis Simple Rules

The study also assessed the sensitivity, specificity and accuracy of the US classification systems and CA-125 in distinguishing benign from malignant adnexal masses. The ROC curve showed that the best cutoff value for CA125 in distinguishing benign from malignant adnexal masses was 42.05 The diagnostic performance of the IOTA SR, O-RADS, and CA-125 is presented in Table 4 and Fig. 2. The findings showed that the AUCs for the IOTA SR, O-RADS, and CA-125 in discriminating benign from malignant adnexal masses were 0.831 (95% CI, 0.788–0.873), 0.804 (95% CI, 0.758–0.849) and 0.812 (95% CI, 0.770–0.884), respectively. In addition, the sensitivity of IOTA SR, O-RADS and CA-125 was 94.42, 94.42 and 80.30%, respectively, and their specificity was 71.74, 66.30 and 82.07%, respectively, while their accuracy was 85.21, 83.00 and 81.02%, respectively.

**Table 4:** Efficacy of IOTA Simple Rules, O-RADS and CA125

	AUC	Sensitivity	Specificity	PPV	NPV	Accuracy
IOTA Simple Rules	0.831 (0.788–0.873)	94.42%	71.74%	83.01%	89.80%	85.21%
O-RADS	0.804 (0.758–0.849)	94.42%	66.30%	80.40%	89.05%	83.00%
CA125	0.812 (0.770–0.884)	80.30%	82.07%	86.75%	74.02%	81.02%

*IOTA* International Ovarian Tumor Analysis, *O-RADS* Ovarian Adnexal Reporting and Data System, *CA 125* Cancer Antigen 125, *AUC* Area under the curve, *PPV* Positive predictive value, *NPV* Negative predictive value

Moreover, ROC analysis showed that a combination of IOTA SR, O-RADS and CA125 had a larger AUC than a combination of IOTA SR and CA125 and a combination of O-RADS and CA125 (0.909, 95% CI = 0.879–0.940 vs. 0.900, 95% CI 0.868–0.932 and 0.891, 95% CI 0.858–0.924; *p* = 0.690 and *p* = 0.440, respectively), as shown in Fig. 1. Additionally, a combination of the three had a significantly higher AUC than IOTA SR, O-RADS and CA125 alone (*p* = 0.004, *p* < 0.001, and *p* < 0.001).

**Fig. 3:**
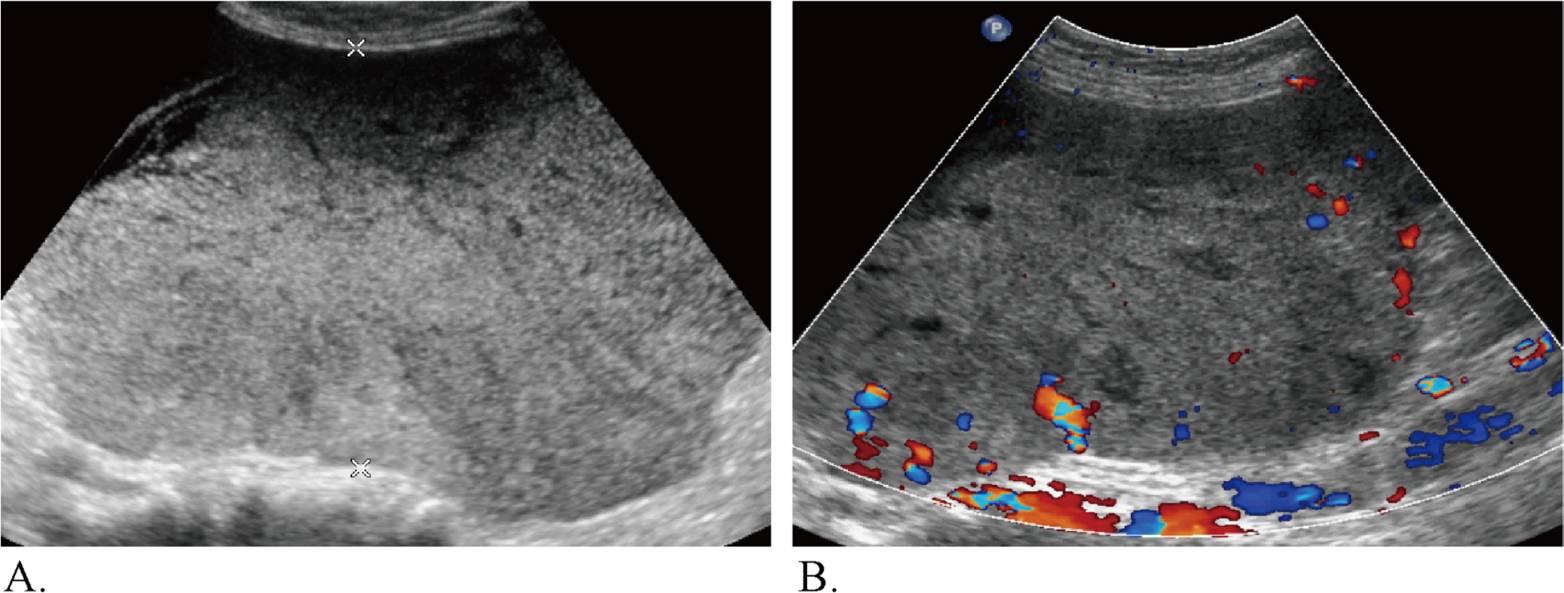
Ultrasound images of a 53-year-old woman whose CA 125 level was 965 U/ml and who had a pathologically proven endometrioid carcinoma. **A** Abdominal grayscale ultrasound showed a 17.6-cm irregular solid tumor component in the right adnexa. **B** A color Doppler ultrasound image showing moderate flow (color score = 3). The lesion was categorized as IOTA SR M1 and O-RADS 5, according to the sonographic findings

**Fig. 4:**
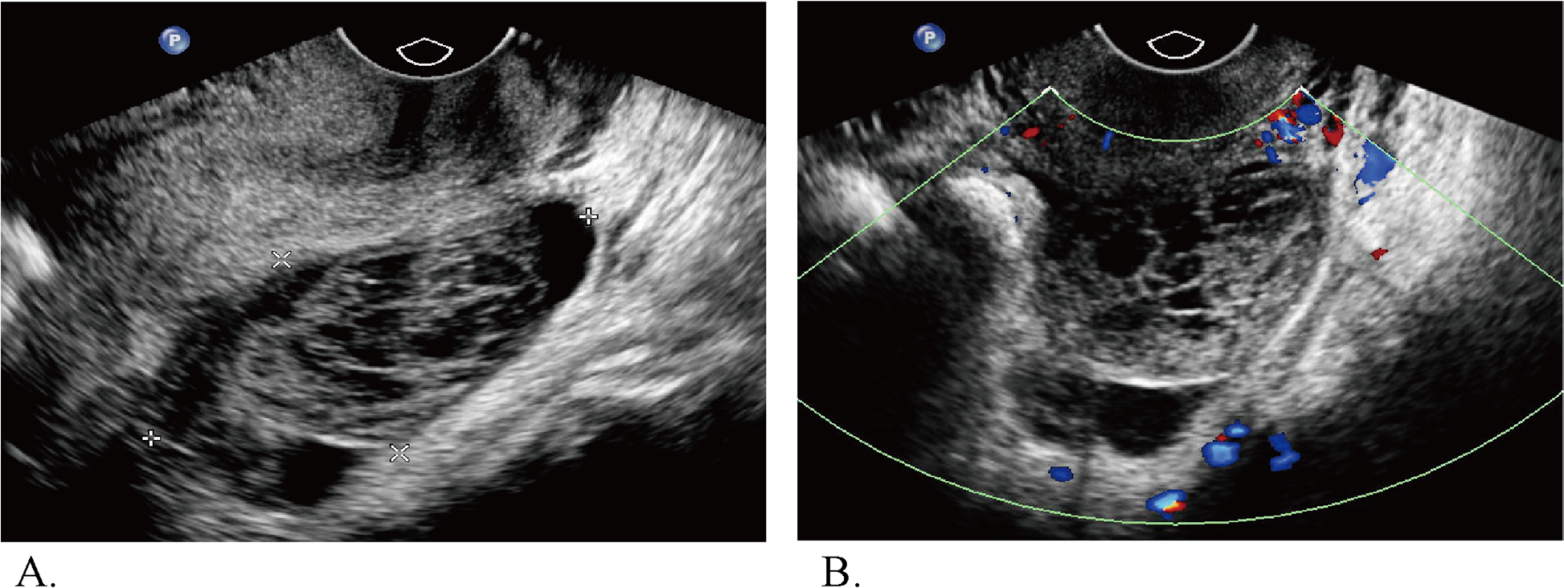
Ultrasound images of a 31-year-old woman with a pathologically proven hemorrhagic cyst and a CA 125 level of 28 U/ml. **A** Transvaginal ultrasound revealed a 6.5-cm multilocular cyst with no solid component in the left adnexa. **B** Color Doppler ultrasound revealed no color flow (color score = 1). The lesion was categorized as IOTA SR B4 and B5 and O-RADS 3 based on the sonographic findings

**Fig. 5:**
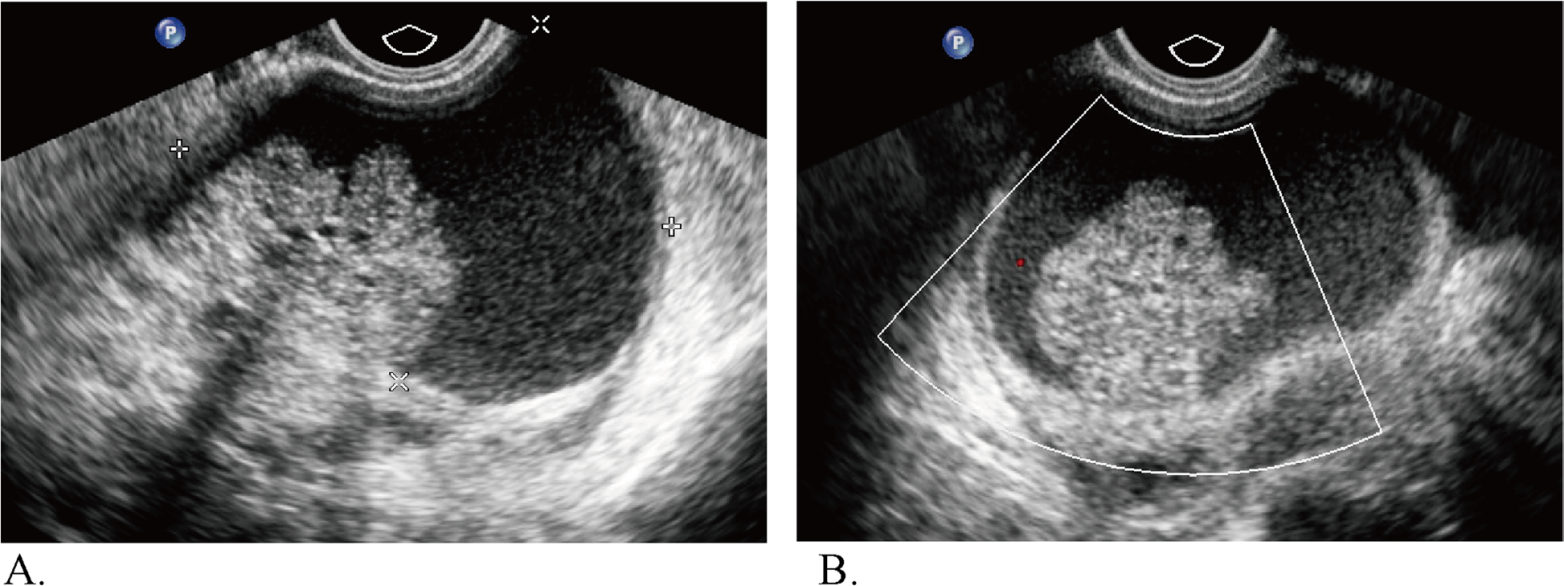
Ultrasound images of a 41-year-old woman with mucinous adenofibroma. The level of CA 125 was 210 U/ml. **A** Transvaginal ultrasound revealed a 7.3-cm complex cystic lesion with a solid component > 7 mm in the right adnexa. **B** Color Doppler ultrasound revealed no color flow (color score = 1). The lesion was categorized as IOTA SR inconclusive and O-RADS 4, according to US category systems

The representative cases in this study are shown in Figs. 3, 4, and 5.

### Inter-reader agreement

The inter-reader agreement for classifying lesion categories by IOTA SR was 0.73 (*p*<0.001), and the two radiologists showed good agreement (detailed categorization is shown in Supplementary Tables 1 and 3). The inter-reader agreement of the O-RADS was 0.62 (*p*<0.001), and the two radiologists showed good agreement (detailed categorization is shown in Supplementary Tables 2 and 3).

## Discussion

The purpose of this study was to investigate the efficiency of IOTA SR, O-RADS, and CA125 in discriminating benign and malignant adnexal masses. The findings showed that IOTA SR was more effective than O-RADS and CA125 in the preoperative diagnosis of adnexal tumors. While the US classification system had a relatively satisfactory diagnostic accuracy in differentiating benign from malignant adnexal masses, US alone, even with expert examiners, is not sufficiently reliable in the detection of malignant tumors [9]. Additionally, the experience of a sonographer is often the primary limiting factor in the evaluation of ovarian masses, as it may affect the diagnostic performance of ultrasound. Moreover, since the levels of CA125 were reported to increase in both benign lesions and other types of ovarian tumors, CA125 may not be effective in discriminating benign from malignant tumors [19]. To the best of our knowledge, few studies have investigated whether a combination of CA125 and US classification increases the preoperative accuracy of differentiating benign from malignant adnexal masses. The results from the present study showed that a combination of serum CA125 data and ultrasound features increased the diagnostic accuracy of differentiating benign from malignant ovarian tumors. More specifically, the findings revealed that a combination of IOTA SR, O-RADS and CA125 had a significantly better diagnostic performance than the individual strategies. However, there was no significant difference between a combination of two methods (IOTA SR & CA125 and O-RADS & CA125) and a combination of all three strategies. This study therefore showed that IOTA SR or O-RADS combined with CA125 can be used in the preoperative differentiation of benign from malignant lesions in place of a combination of the three strategies.

Extensive research has been conducted on IOTA SR. For instance, a prospective study on the external validation of IOTA SR showed that the overall sensitivity and specificity of the method were 94.3 and 94.9%, respectively [1]. Moreover, a meta-analysis including 19,674 adnexal tumors indicated that the pooled sensitivity and specificity of IOTA SR were 93.0 and 80.0%, respectively [13]. However, simple rules could be used in 76–89% of tumors due to the inconclusive patients, and the patients therefore needed further expert consultation. Nonetheless, several reports have shown that a combination of ultrasound features and biomarkers is more accurate in predicting malignancy in ovarian cancer [8]. For example, a recent study reported that a combination of CA-125 and IOTA SR had a better diagnostic value in differentiating between malignant and benign ovarian tumors [14], consistent with the findings from the present study. Rapeepat et al also showed that the IOTA SR had a higher diagnostic efficiency than the risk of malignancy index (RMI) in discriminating benign from malignant ovarian masses [4].

In addition, O-RADS provides standardized descriptors and definitions of the US characteristics of normal ovaries and ovarian lesions. Nonetheless, few studies have been conducted on O-RADS. A previous analysis of 1054 adnexal masses indicated that the AUC of O-RADS was 0.960 (95% CI, 0.947–0.971) [6]. Moreover, a recent study compared O-RADS to IOTA SR in 609 women, and the results revealed that O-RADS had a higher sensitivity than IOTA SR in the diagnosis of adnexal masses [5]. The present study showed that IOTA SR had a higher AUC than O-RADS, contrary to previous findings. This may be associated with the proportion of benign and malignant ovarian masses in previous and current studies. Notably, the present study had more malignant ovarian masses, while the previous report had more benign ovarian masses. Furthermore, this was a retrospective study where the assessment of color flow in the masses was limited.

The main strengths of the present study are that a relatively large number of patients were enrolled, and nearly all histological subtypes of ovarian cancers were included. However, the study had a few limitations, including its retrospective design. First, ultrasound evaluation was initially performed by examiners with different levels of experience, which may have affected image storage to some extent. The retrospective nature of the study may also have led to selection bias given that only patients scheduled for surgery were recruited. Only CA125 was used out of all other tumor markers, which should also be a limitation of the study. Furthermore, only a few women underwent transvaginal ultrasound examination in the present study, since the transvaginal ultrasound was closer to the ovary lesion than traditional ultrasound.

## Conclusion

In summary, this study revealed that a combination of the IOTA SR or O-RADS and CA125 data is more accurate in classifying adnexal masses as either benign or malignant. Therefore, combining the serum levels of CA125 with ultrasound features may have a more significant diagnostic value than individual approaches in discriminating ovarian tumors.

## Supplementary Information

**Additional file 1.**

## Data Availability

All data generated or analyzed during this study are included in this published article.
