# Supplementary material for: Efficacy of IOTA simple rules, O-RADS, and CA125 to distinguish benign and malignant adnexal masses
Source: J Ovarian Res. 2022 Jan 23;15:15. doi: 10.1186/s13048-022-00947-9 (PMC8785584; doi:10.1186/s13048-022-00947-9)
Supplement: Supplementary file 1 — Additional file 1. [file 13048_2022_947_MOESM1_ESM.doc]

Supplementary Table 1. The inter-reviewer agreement of IOTA SR classification for diagnosis of adnexal masses.

|  |  | Observer 2 | | |  |
| --- | --- | --- | --- | --- | --- |
|  |  | Benign | Malignant | Inconclusive | Total |
| Observer 1 | Benign | 125 | 5 | 17 | 147 |
|  | Malignant | 13 | 209 | 7 | 229 |
|  | Inconclusive | 10 | 20 | 47 | 77 |
|  | Total | 148 | 234 | 71 | 453 |

Supplementary Table 2. The inter-reviewer agreementof O-RADS classification for diagnosis of adnexal masses.

|  |  | Observer 2 | | | |  |
| --- | --- | --- | --- | --- | --- | --- |
|  |  | O-RADS2 | O-RADS3 | O-RADS 4 | O-RADS5 | Total |
| Observer 1 | O-RADS 2 | 52 | 6 | 18 | 2 | 78 |
|  | O-RADS 3 | 0 | 32 | 24 | 3 | 59 |
|  | O-RADS 4 | 0 | 5 | 90 | 39 | 134 |
|  | O-RADS 5 | 0 | 0 | 20 | 162 | 182 |
|  | Total | 52 | 43 | 152 | 206 | 453 |

Supplementary Table 3. Frequency distributions of IOTA SR and O-RADS for 453 adnexal masses stratified by system and reviewer

| Category | Characteristic | Reviewer 1 | Reviewer 2 | κ value |
| --- | --- | --- | --- | --- |
| IOTA |  |  |  | 0.73 |
| Benign | Only B features | 147(32.4) | 148(32.7) |  |
| Malignant | Only M features | 229(50.6) | 234(51.7) |  |
| Indeterminate | No features or both M and B features | 77(17.0) | 71(15.6) |  |
| O-RADS |  |  |  | 0.62 |
| O-RADS2 | ＜1% risk of malignancy | 78(17.2) | 52(11.5) |  |
| O-RADS3 | 1% -＜10% risk of malignancy | 59(13.0) | 43(9.5) |  |
| O-RADS4 | 10%-50% risk of malignancy | 134(29.6) | 152(33.5) |  |
| O-RADS5 | ≥50% risk of malignancy | 182(40.2) | 206(45.5) |  |

Data are number of adnexal masses. Data in parentheses are percentages

IOTA = International Ovarian TumorAnalysis, O-RADS = Ovarian-Adnexal Reporting and Data System.
